# Changes in Bihemispheric Structural Connectivity Following Middle Cerebral Artery Infarction

**DOI:** 10.3390/jpm12010081

**Published:** 2022-01-10

**Authors:** Dae Hyun Kim, Hyunkoo Kang

**Affiliations:** 1 Department and Research Institute of Rehabilitation Medicine, Yonsei University College of Medicine, Seoul 03722, Korea; 2 Veterans Health Service Medical Center, Department of Radiology, Seoul 05368, Korea; knroo@bohun.or.kr

**Keywords:** stroke, recovery, lateralization, structural connectivity

## Abstract

This study investigated the changes in the structural connectivity of the bilateral hemispheres over time following a middle cerebral artery infarction. Eighteen patients in the subacute group and nine patients in the chronic group with mild upper extremity motor impairment (Fugl-Meyer motor assessment score for the upper limb > 43) following middle cerebral artery infarction were retrospectively evaluated in this study. All the patients underwent T1-weighted and diffusion tensor imaging. Tract-based statistical analyses of fractional anisotropy were used to compare the changes in the bilateral structural connectivity with those of age-matched normal controls. The corticospinal tract pathway of the affected hemisphere, corpus callosum, and corona radiata of the unaffected hemisphere had decreased structural connectivity in the subacute group, while the motor association area and anterior corpus callosum in the bilateral frontal lobes had increased structural connectivity in the chronic group. The bilateral hemispheres were influenced even in patients with mild motor impairment following middle cerebral artery infarction, and the structural connectivity of the bilateral hemispheres changed according to the time following the stroke.

## 1. Introduction

Motor impairment is a common symptom following a stroke, which can reduce the quality of life. In the subacute and chronic phase following a stroke, it shows a prevalence of 78% and 67%, respectively [1]. Cerebral damage after a stroke is followed by a certain degree of motor recovery, which contributes to neuronal reorganization [2,3]. This neuronal reorganization may be managed by a number of rehabilitative treatments, including physical therapy, non-invasive brain stimulation, and pharmacological agents [3,4]. Understanding neuronal reorganization could help target treatment and promote motor recovery following a stroke. Based on evidence from animal models, it is now well known that a stroke triggers molecular cascades related to the reorganization and repair mechanisms [5,6]. Since the tools available for studying the reorganization of the human brain are very different from those available in animals, the reorganization process following a stroke is still unclear in humans.

Functional neuroimaging studies of patients with stroke have demonstrated the underlying neuronal processes driving the recovery of motor function following a stroke [7,8,9,10]. The neural activity in the cortical motor areas during upper limb movements is enhanced in both the hemispheres in the subacute phase and subsequently decreases in the motor areas of the unaffected hemispheres in the chronic phase following a stroke [8,9,11]. Notably, the recovery process of upper limb motor function following a stroke includes various structural and functional changes in the affected and unaffected hemispheres. However, previous studies were limited to the functional changes in the bilateral hemispheres. No studies have demonstrated the bilateral hemispheric structural connectivity changes according to the time following the stroke. Therefore, this study aimed to investigate the changes in the structural connectivity of the bilateral hemispheres in patients with mild upper limb motor impairments compared to that in a normal control group, according to the subacute and chronic phase following a stroke.

## 2. Methods

### 2.1. Patient Groups

In this study, we retrospectively reviewed the medical records of 384 patients who underwent diffusion-tensor imaging (DTI) between May 2015 and May 2020. The inclusion criteria were (a) first ever unilateral stroke, as confirmed by magnetic resonance imaging (MRI), (b) middle cerebral artery infarction, (c) mild upper extremity motor impairment (Fugl-Meyer motor assessment score for the upper limb > 43) [12], (d) <3-months cerebral infarction for the subacute group or >1-year cerebral infarction for the chronic group, and (e) DTI and motor function evaluation within a 1-week interval. The exclusion criterion included any coexisting neurological disease that could influence the upper limb motor function. Eighteen patients in the subacute group and nine patients in the chronic group met the criteria for this retrospective study. All patients in both groups were men owing to the veteran hospital’s characteristics. The characteristics of the subacute and chronic groups were as follows: the mean (standard error) ages were 72.0 (0.9) and 76.7 (2.9) years, the lesion volumes were 13.8 (8.3) and 10.9 (6.2) cm^3^, and the times following the stroke were 42.8 (6.1) and 2147.4 (833.0) days, respectively. There were no significant differences between the groups, except for the time following the stroke (Table 1). The study protocol was approved by our Institutional Research Ethics Committee for Human Subjects, which waived the requirement for informed consent owing to the retrospective study design.

### 2.2. Healthy Control Group

Age-matched male participants were randomly selected from the Cambridge Centre for Ageing and Neuroscience cohort [13]. We included 36 healthy controls for the subacute group and 18 healthy controls for the chronic group, resulting in a patient-to-control ratio of 1:2.

### 2.3. MRI Acquisition and Analysis

**Figure 1:**
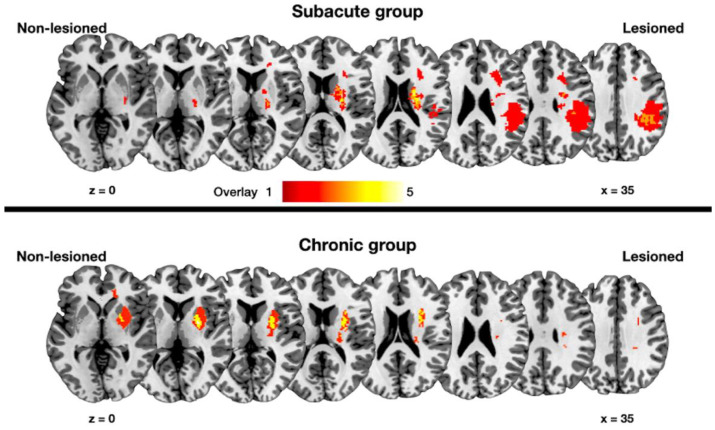
Lesion overlapping maps of the subacute and chronic groups. The color scale indicates the number of overlapping lesions across the patients. Lesioned: Lesioned hemisphere; Non-lesioned: non-lesioned hemisphere; z: *z*-axis in the Montreal Neurological Institute space.

All MRI scans were acquired using a 3-T MR scanner (Siemens, Erlangen, Germany) with a 20-channel head coil. High-resolution three-dimensional (3D) T1-weighted images and DTI scans were obtained. Detailed parameters of the T1-weighted images and DTI are shown in Table 2. For lesion volume measurements, a neuroradiologist constructed each patient’s lesion on the T1-weighted images in the native space using the 3D slicer software (https://www.slicer.org accessed on 20 December 2017). For analysis, the DTI was registered to the corresponding b = 0 image with an affine transformation to amend the distortions attributed to the eddy currents (FSL 5.0.10; http://www.fmrib.ox.ac.uk/fsl). Fractional anisotropy (FA) maps were subsequently constructed in the native space. T1-weighted images and DTI of 13 right hemispheric lesions were flipped to align the corresponding lesion area in the left hemisphere. Voxel-based lesion symptom mapping was employed to compare the two groups for identifying the lesion effect. Voxel-based lesion symptom mapping showed no significant lesion differences between the groups (Figure 1). Trac-based statistical analysis of the FA maps was performed using the FMRIB Software Library [14]. FA maps were non-linearly registered to the Montreal Neurological Institute space. The registered FA maps for the patients and controls were projected onto the mean FA skeleton, representing the centers of the common fiber bundles. The resulting maps were fed into voxel-wise statistical analyses to compare patient and age-matched control groups. The statistical analyses were performed with the threshold-free cluster enhancement nonparametric permutation test, using 5000 Monte Carlo simulations [14]. The statistical threshold was set at a corrected *p*-value of less than 0.05. The group characteristics were compared using the two-sample *t*-test with R version 3.5.2 (R Foundation for Statistical Computing, Vienna, Austria).

## 3. Results

### 3.1. Changes in the Structural Connectivity in the Subacute Group

**Figure 2:**
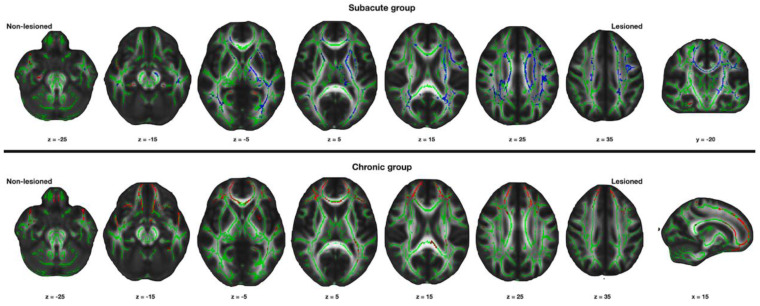
Results of the tract based statistical analysis. Red areas indicate increased values of fractional anisotropy in the patient group compared with the age-matched control group. Blue areas indicate decreased values of fractional anisotropy in the patient group compared with the age-matched control group. x, y, z: x-, y-, *z*-axis in Montreal Neurological Institute space. Statistical threshold: corrected *p* < 0.05.

Compared with the control group, the patient group had significantly decreased FA values in the affected hemisphere, mainly in the corticospinal tract pathway and corpus callosum. The FA values of the corona radiata and the corpus callosum of the unaffected hemisphere were also significantly decreased in the patients compared with the control group (corrected *p* < 0.05, Figure 2). 

### 3.2. Changes in the Structural Connectivity in the Chronic Group

In the chronic group, the FA values were significantly increased mainly in the motor association brain areas of the bilateral frontal lobes compared with that in the control group. The FA value did not significantly decrease in the brain region in the patient group compared with the control group (corrected *p* < 0.05, Figure 2). 

## 4. Discussion 

In this study, we demonstrated that the structural connectivity of the bilateral hemispheres changed from the subacute to the chronic phase following a stroke. The parallel movement of water molecules to the fiber tracts correlates with the FA values [15]. A higher FA value indicates fiber tract integrity, which corresponds to increased structural connectivity [16,17]. Structural connectivity decreased in the bilateral hemispheres during the subacute phase. However, structural connectivity increased bilaterally, mainly in the frontal lobe during the chronic phase following a stroke.

A focal infarct can induce widespread changes in the connected and distant brain areas. Increased functional activity in the bilateral hemisphere and reduced laterality is one of the main reorganization processes in the subacute phase following a stroke [2,11,18,19,20]. Previous DTI studies also reported the positive association between increased structural connectivity and motor function following a stroke [21,22]. The bihemispheric activation can be seen as a passive event, reflecting a reduced interhemispheric inhibition resulting from the stroke lesion [2,11,23]. However, the bihemispheric activation differs from our results of decreased structural connectivity in the subacute group. The structural connectivity reflects the structural changes following stroke instead of the cortical activity [2,11]. The decrease in the structural connectivity may result from diaschisis. Diaschisis refers to the reduced activity in the uninjured brain areas that have rich connections with the injured brain areas [24,25]. The corona radiata, which passes through the corticospinal tract, and the corpus callosum were mainly the decreased brain areas in the unaffected hemisphere. The motor-related brain regions and the corticospinal tract have rich connections through the corpus callosum; diaschisis in the subacute phase following stroke may have caused a decrease in the bihemispheric structural connectivity in this study. 

The mechanism of the changes in the unaffected hemisphere following a stroke is still unclear. Previous functional MRI studies have demonstrated that better motor recovery is based on the activation of the motor areas in the affected hemisphere rather than in the unaffected hemisphere [2,11,20]. However, other studies reported that the activation of the motor cortex in the affected hemisphere had no relation to the degree of motor recovery [26]. The results of this study also showed the bilateral increase in the structural connectivity mainly in the frontal areas. The reorganization process is not limited to the motor area in the affected hemisphere [27,28]. Several factors, such as severity, location, and duration following a stroke influence the unaffected hemisphere in the recovery following a stroke [29]. Since the stroke lesion was limited to the motor associated areas in the frontal lobe, including the basal ganglia and corticospinal tract pathway in our chronic group, the structural connectivity increased in the motor association areas in the bilateral frontal lobes. Further prospective studies are warranted to investigate the inter-individual differences affecting the reorganization process following a stroke. 

Our study has several limitations. First, this is a retrospective study. Second, this study has a small sample size and heterogenous patients. However, this may be the first study to analyze the changes in structural connectivity compared with that in normal controls according to the time following the stroke. 

## 5. Conclusions

Bilateral decrease in structural connectivity may result from diaschisis in the subacute phase following a stroke. The unaffected hemisphere increases the structural connectivity in the recovery process of the chronic phase following a stroke.

## Figures and Tables

**Table 1:** General characteristics of the included patients.

Group	Age (Years)	Duration	FMUL	Lesion Volume (cm^3^)
Subacute	75.8	12	48	6.0
Subacute	67.2	13	59	5.8
Subacute	75.6	16	64	1.8
Subacute	68.9	17	64	1.3
Subacute	69.7	17	47	6.1
Subacute	75.8	18	44	2.2
Subacute	70.2	19	66	153.2
Subacute	72.3	29	43	0.2
Subacute	70.8	32	63	15.1
Subacute	68.6	46	55	25.6
Subacute	73.3	52	57	3.5
Subacute	71.9	62	63	6.5
Subacute	71.4	62	47	2.5
Subacute	70.9	67	66	1.6
Subacute	81.5	72	52	5.2
Subacute	73.4	76	45	3.1
Subacute	64.0	78	57	3.7
Subacute	74.3	82	64	4.6
Chronic	74.4	383	60	9.9
Chronic	68.9	454	66	0.8
Chronic	66.8	558	63	57.4
Chronic	73.5	643	63	3.3
Chronic	94.3	1237	63	19.8
Chronic	72.8	1428	64	0.6
Chronic	77.1	2911	63	3.4
Chronic	86.2	3649	60	1.1
Chronic	75.9	8064	64	1.7

Subacute: subacute group, Chronic: chronic group, FMUL: Fugl-Meyer motor assessment score for the upper limb.

**Table 2:** Magnetic resonance imaging data acquisition.

Parameter	T1	DTI
Matrix	256 × 256	112 × 112
Field of view (mm^2^)	230 × 230	224 × 224
Repetition time (ms)	1900	9700
Echo time (ms)	2.57	92.00
Slice thickness (mm)	1	2
Flip angle (°)	9	9

Other parameters were as follows: DTI, 30 directions, b = 1000 s/mm^2^. T1: T1-weighted imaging. DTI: diffusion-tensor imaging.

## Data Availability

The data presented in this study are available on request from the corresponding author. The data are not publicly available due to privacy.
